# The Occurrence of Early-Onset Neonatal Streptococcus pneumoniae Infection as a Result of Intrapartum Infection

**DOI:** 10.7759/cureus.32613

**Published:** 2022-12-16

**Authors:** Hiroyuki Yamada, Yusuke Kurooka, Masafumi Fujimoto, Masaaki Ueda, Toshinori Minato

**Affiliations:** 1 Department of Pediatrics, Toyooka Public Hospital, Toyooka, JPN

**Keywords:** intrapartum infection, streptococcus pneumoniae, congenital pneumonia, early-onset neonatal infection, neonatal sepsis

## Abstract

Early-onset neonatal infection caused by *Streptococcus pneumoniae* occurs rarely but has a high mortality rate. Due to the low detection rate of *S. pneumoniae* in maternal vaginal cultures, administering prophylactic antibiotics for *S. pneumoniae *to mothers before delivery is challenging. Herein, we present the case of a male newborn who was born at 38 weeks of gestation. The vaginal cultures of his mother before delivery did not reveal the presence of group B streptococcus (GBS) and *S. pneumoniae*. The newborn experienced respiratory distress six hours after birth and was diagnosed with congenital pneumonia. He was successfully treated with an artificial ventilator and antibiotics. The nasal cavity, external ear canal, and transtracheal tube sputum cultures of the neonate and the vaginal cultures of his mother were positive for *S. pneumoniae* serotype 3. This case indicates the occurrence of congenital *S. pneumoniae* infection as a result of intrapartum infection and highlights the necessity to consider *S. pneumoniae* as a causative agent of early-onset neonatal infection.

## Introduction

The incidence of early-onset neonatal sepsis (EONS) caused by *Streptococcus pneumoniae* has been reported to be approximately 1% [1]; however, the mortality rate is reported to be as high as 50% [2]. All documented neonatal deaths due to EONS caused by *S. pneumoniae* occurred within 36 hours of presentation [2]. Invasive *S. pneumoniae* is considered to be transmitted from mothers to neonates during delivery or intrauterine infections in mothers; however, the route of transmission of *S. pneumoniae* from mothers to neonates cannot be determined due to the small number of reported cases. Furthermore, maternal vaginal cultures to detect group B streptococcus (GBS) are often performed in the later stage of pregnancy. In neonates with early-onset invasive infections, early intervention is required for GBS and *S. pneumoniae*. Herein, we present a case of early-onset neonatal infection caused by *S. pneumoniae* where the patient was successfully treated with antibiotics and respiratory management and he recovered completely.

## Case presentation

A male newborn weighing 3,164 g was delivered vaginally at 38 weeks of gestation and had an Apgar score of 8 and 10 at one and five minutes, respectively. His mother was 30 years old, gravida 1 and para 0, and had an uneventful pregnancy; however, the agenesis of the corpus callosum was revealed via fetal ultrasonography at 26 weeks of gestation. Maternal vaginal culture was negative for GBS at 35 weeks of gestation. The mother did not have an intrapartum high fever. Before the delivery, fetal tachycardia was not detected. The newborn was admitted to the neonatal intensive care unit for monitoring abnormalities of the central nervous system and anomalies associated with the agenesis of the corpus callosum. He presented with respiratory distress six hours after birth. He was pale, his respiratory rate increased to 100 breaths per minute with grunting, and his oxygen saturation (SpO_2_) dropped to 90%-95% (room air). His vital signs were as follows: temperature, 38.6°C; heart rate, 150-160 beats per minute; and blood pressure, 56/32 mmHg. Venous blood gas analysis before the initiation of artificial ventilation revealed that pH was 7.197, partial pressure of carbon dioxide (pCO_2_) level was 62.3 mmHg, bicarbonate (HCO_3_) level was 23.3 mmol/L, base excess was -6.6 mmol/L, and lactate level was 3.8 mmol/L. Nasal continuous positive airway pressure was initiated but was ineffective. Therefore, the patient was intubated and supported by an artificial ventilator.

**Figure 1:**
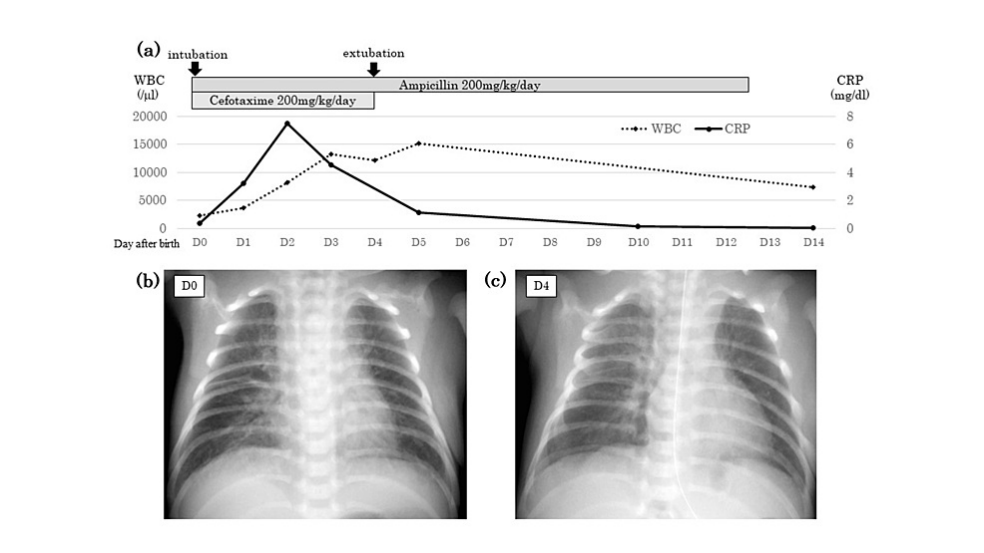
Laboratory test results and chest radiograph findings. (a) The graph indicates changes in the white blood cell count and C-reactive protein level, as well as the antibiotics administered. (b) Chest radiograph on admission (zero days old). The radiograph shows noticeable consolidation in the right middle and lower lung fields. (c) Chest radiograph at four days of life showing improvement in the consolidation in the lung fields. As the neonate’s respiratory condition improved, he was extubated on the same day. WBC, white blood cell; CRP, C-reactive protein

Other initial laboratory findings included leukocytopenia (2,300/µL), neutropenia (460/µL), normal platelet count (21.3 × 104/µL), and normal C-reactive protein level (0.34 mg/dL; normal range: <0.5 mg/dl) (Figure 1a). Chest radiographs revealed consolidation in the right middle and lower lung fields (Figure 1b). Cerebrospinal fluid (CSF) analysis revealed no leukocytosis and normal protein and glucose levels. The cultures from the nasal cavity, external ear canal, and transtracheal tube sputum were positive for *S. pneumoniae*. However, the cultures from blood and CSF were reported negative for *S. pneumoniae*. The newborn was diagnosed with early-onset neonatal infection with pneumonia, and ampicillin (200 mg/kg/day) and cefotaxime (200 mg/kg/day) were administered starting 12 hours after birth. Following antibiotic administration, his vital signs, laboratory parameters, and chest radiographic findings improved. He was managed with an artificial ventilator in the pressure control-synchronized intermittent mandatory ventilation (PC-SIMV) mode with positive inhaled pressure (PIP) of 14 mmHg, positive end-expiratory pressure (PEEP) of 5 mmHg, respiratory rate of 60 per minute, inspiratory:expiratory (I:E) ratio of 1:1, and fraction of inspired oxygen (FiO_2_) of 0.30. He was extubated on the fourth day of life (Figure 1a-1c). As *S. pneumoniae* is sensitive to ampicillin, the medication was continued from the fifth day to the 12th day of life. He received parenteral nutrition while intubated, but he was soon able to feed orally after extubation. The patient eventually recovered without any complications. Meanwhile, the vaginal culture of his mother was positive for *S. pneumoniae*. The serotype of *S. pneumoniae* detected in the samples from the neonate, and that in the vaginal culture of the mother was later found to be identical (serotype 3). At eight months, the patient showed normal growth and development according to his age.

## Discussion

The patient reported herein was diagnosed with early-onset neonatal pneumonia caused by *S. pneumoniae*. Intrapartum vertical infection was suspected to be the mode of transmission in this case. Neonatal infection caused by *S. pneumoniae* is rare; among 270 EONS cases reported in a previous study, only two had blood cultures positive for *S. pneumoniae* [1]. However, in a previous study by Oeser et al., polymerase chain reaction (PCR) detected *S. pneumoniae* in the blood samples of 91/203 (45%) infants with EONS with negative blood cultures. In addition, among PCR-positive EONS infants, *S. pneumoniae* was detected in 21 cases (18%) [3]. Therefore, the proportion of EONS caused by *S. pneumoniae* may be higher than that reported in previous studies. *Streptococcus pneumoniae* has been reported to transmit from mothers to neonates through the placenta and amniotic cavity in addition to intrapartum infection [2]. In this case, although the blood culture results were negative for the infant, *S. pneumoniae* serotype 3 was detected in the nasal cavity, external ear canal, and intratracheal sputum samples of the infant and vaginal culture of the mother, suggesting intrapartum infection.

In Japan, vaginal cultures are exclusively performed in the third trimester of pregnancy to detect GBS. In addition, antenatal prophylactic antibiotic therapy for the prevention of neonatal invasive GBS infection has been established. However, administering prophylactic antibiotics for *S. pneumoniae* to mothers before delivery is difficult due to the low detection rate of *S. pneumoniae* in maternal vaginal cultures [4,5]. Furthermore, the timing of vaginal cultures, as well as the effectiveness of prophylactic administration of antibiotics, should be further investigated because various routes of transmission are suspected. Nevertheless, vaginal cultures before delivery should be performed because the mortality rate of neonatal *S. pneumoniae* infection is 50% [2]. Both penicillin and third-generation cephalosporin or gentamycin should be administered when treating neonatal sepsis to manage infections caused by GBS, *Escherichia coli*, and *Listeria*. In addition, this regimen may also be effective in treating penicillin-resistant *S. pneumoniae* infection. The results of maternal vaginal cultures before delivery facilitate early interventions for neonates with early-onset invasive infections.

## Conclusions

In neonates diagnosed with early-onset infection, *S. pneumoniae* and GBS should be considered causative pathogens. Since early-onset *S. pneumoniae* infection in neonates is considered to occur via intrapartum infection, it is reasonable to administer antibiotics effective against *S. pneumoniae* in cases showing the presence of *S. pneumoniae* in maternal vaginal cultures in the third trimester of pregnancy. Early pathogen identification and treatment may lead to a better prognosis in patients with early-onset neonatal infection.
